# Dual-band electrochromism in two-dimensional layered TT-Nb_2_O_5_

**DOI:** 10.1093/nsr/nwaf266

**Published:** 2025-07-08

**Authors:** Wu Zhang, Delia J Milliron

**Affiliations:** McKetta Department of Chemical Engineering, University of Texas at Austin, USA; McKetta Department of Chemical Engineering, University of Texas at Austin, USA

Electrochromism is the reversible change of optical properties in response to the application of an electrochemical stimulus [1]. For application in smart windows, dual-band electrochromism enables the independent modulation of visible and near-infrared (NIR) light transmittance [2], which can enhance the energy efficiency of buildings by managing solar heat gain and daylighting.

The first demonstration of dual-band electrochromism was achieved by introducing tin-doped indium oxide (ITO) nanocrystals into a glassy niobium oxide matrix [3]. In this composite film, the transmittance of visible and NIR light can be independently modulated based on the applied potential, with the ITO nanocrystals blocking NIR light via collective excitation of delocalized electrons, i.e. localized surface plasmon resonance, and the niobium oxide blocking visible light through absorption by localized electrons, i.e. polarons formed when the atomic structure locally rearranges around inserted lithium ions. More recently, dual-band electrochromism has been described in single-component thin films that exhibit two mechanisms of optical response, e.g. plasmonic and polaronic behaviors, depending on the applied electrochemical potential [4,5]. Interestingly, such single-component dual-band performance has been reported in a layered crystalline phase of niobium oxide, emphasizing the importance of atomic scale structure in determining the optical response, as well as the electrochemical properties of the material [6,7].

**Figure 1.**
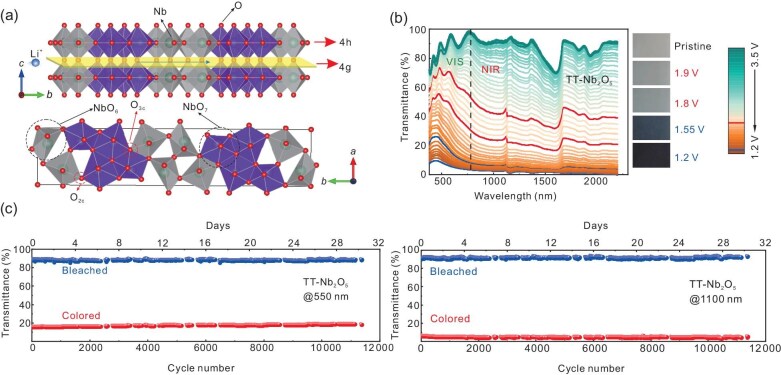
(a) A $1 \times 1 \times 2$ superstructure of TT-Nb$_2$O$_5$ is shown, viewing along the *a* axis and *c* axis. The schematic illustration of the lithium ion diffusion layer (4g) is marked in yellow with distorted octahedra (NbO$_6$) and pentagonal bipyramids (NbO$_7$) shown in gray and purple. The O$_{\rm 2c}$ and O$_{3{\rm c}}$ refer to two distinct oxygen coordinations in the 4h layer, which are suggested by density functional theory as the sites for lithium localization. (b) The optical transmittance variation of TT-Nb$_2$O$_5$ thin films. The red bold curve represents the cool mode (1.90 and 1.80 V), while the blue bold curve represents the dark mode (1.55 and 1.20 V), along with the corresponding digital photographs. (c) The cycling performance of TT-Nb$_2$O$_5$ thin films monitored at 550 and 1100 nm. Reproduced from [8] with permission.

In a recent study published in *National Science Review*, Professor Rui-Tao Wen’s group at Southern University of Science and Technology reported that dual-band electrochromism can be realized in a high-temperature phase of niobium oxide, specifically thin films of the two-dimensional (2D) layered TT-Nb$_2$O$_5$ (pseudohexagonal) phase (Fig. 1a) [8]. Unlike nanocrystal-based films that are solution processed, the TT-Nb$_2$O$_5$ films were fabricated using reactive direct current magnetron sputtering onto conductive quartz substrates, followed by thermal annealing. Unlike the as-deposited amorphous niobium oxide films or orthorhombic T-Nb$_2$O$_5$ films realized by annealing at $600\, ^\circ {\rm C}$, the TT-Nb$_2$O$_5$ thin films produced after $850\, ^\circ {\rm C}$ annealing exhibit pronounced dual-band electrochromism. The transparent films (Fig. 1b, 3.5 V in green) initially modulate primarily in the NIR region upon application of a reducing potential down to 1.8 V versus Li/Li$^{+}$ (red). When the potential decreases to 1.2 V, the visible transmittance and NIR transmittance drop significantly (blue). Density functional theory calculations were used to rationalize the optical response, finding that strain due to lithium ion insertion instigates a change in the electronic structure. The Nb 3D-derived conduction band is predicted to split into sub-bands and gradually shift, while the Fermi level rises due to band filling. The changing density of states makes new optical transitions available, causing absorption to occur at different wavelengths depending on the extent of lithiation. These changes are accompanied by a sequential, partial reduction of the niobium from $5^{+}$ to $4^{+}$ and eventually $3^{+}$, as detected by X-ray photoelectron spectroscopy.

Structurally, TT-Nb$_2$O$_5$ possesses a 2D layered architecture that provides open pathways for lithium ion transport, enabling rapid diffusion with minimal structural distortion. The films demonstrate rapid switching kinetics and exceptional cycling stability, with no observable degradation after over 10 000 cycles (Fig. 1c).

In summary, this paper reports dual-band electrochromism in 2D layered TT-Nb$_2$O$_5$. The electronic structure changes occurring with progressive lithiation offer a route for realizing spectral control as a function of electrochemical potential, distinct from the localized surface plasmon resonance observed in nanocrystals. These insights open the door to new strategies for customizing the optical response of switchable coatings in a range of applications.
